# Multiple Unusual Distant Metastasis in TENIS: Comparative Evaluation of [ ^18^ F]FDG and [ ^68^ Ga]-FAPI-04 PET-CT Uptake Patterns at Metastatic Sites

**DOI:** 10.1055/s-0045-1814419

**Published:** 2025-12-23

**Authors:** Madhava Reddy Mali, Priyanka Verma, Sandip Basu

**Affiliations:** 1 Radiation Medicine Centre, Bhabha Atomic Research Centre, Tata Memorial Centre Annexe, Mumbai, Maharashtra, India; 2 Homi Bhabha National Institute, Mumbai, Maharashtra, India

**Keywords:** thyroid cancer, TENIS, unusual distant metastasis, ^18^ F-FDG PET/CT, [ ^68^ Ga]-FAPI-04 PET/CT

## Abstract

A relatively uncommon occurrence of multiple unusual distant metastasis in a patient with thyroglobulin-elevated negative iodine scintigraphy (TENIS) (subcutis, skeletal muscle, kidney, and pericardium) is presented. A 65-year-old man with papillary thyroid carcinoma post two cycles of radioactive iodine therapy on subsequent follow-up showed elevated serum thyroglobulin (>300 ng/mL) and negative iodine scintigraphy (TENIS). [18F]-fluorodeoxyglucose (FDG) positron emission tomography (PET)/computed tomography (CT) for disease restaging revealed extensive metastatic disease involving cervical and mediastinal nodes, bones, liver, subcutis, skeletal muscle, kidney, and pericardium. [68Ga]-fibroblast activation protein inhibitor (FAPI)-04 PET/CT was done to evaluate for fibroblast activation protein (FAP) expression, and the feasibility of targeted radionuclide therapy showed nil to very low FAP expression at the metastatic sites, except for the few cervical and mediastinal nodes, subcutaneous nodule in the left arm, and L3 vertebral lesion. In addition to illustrate the rare presentation in TENIS, this case demonstrates the superiority of [18F]-FDG PET/CT over FAPI PET/CT in this patient. However, FAPI-PET/CT may be used as a theranostic tool for assessing radionuclide therapy prospects in selected cases.

## Introduction

Thyroglobulin-elevated negative iodine scintigraphy (TENIS) is not an uncommon entity and is encountered in the day-to-day management of differentiated thyroid cancer (DTC). Its incidence is in the range of 2 to 27% from various series reported in the literature. 1 2 Distant metastases and tumor burden confer an unfavorable outcome and poor survival. 2 3 Lungs and skeleton remain the common sites of distant metastasis. Metastasis to multiple unusual distant sites is a relatively rare occurrence. 4 The American Thyroid Association (ATA) 2025 conditionally recommends [18F]-fluorodeoxyglucose (FDG) positron emission tomography (PET)/computed tomography (CT) in TENIS/radio-iodine refractory (RAIR) DTC for staging, prognostication, risk stratification, and treatment response evaluation. 3 Fibroblast activation protein inhibitor (FAPI) PET/CT, a novel theranostic tool, has been showing promising results in TENIS/RAIR-DTC. 5 6 We present an exceptionally rare case of TENIS with multiple unusual distant metastases (UDMs), along with a comparative evaluation of FDG and FAPI PET/CT findings.

## Case Report

A 65-year-old man of locally invasive papillary thyroid carcinoma (PTC) with cervical nodal metastasis underwent surgery for primary and nodal metastasis followed by adjuvant radioactive iodine (RAI) (6.06 GBq). At subsequent follow-up, the patient had an elevated serum thyroglobulin (Tg) (>300 ng/mL) and negative iodine scintigraphy (TENIS). Ultrasonography revealed subcentimetric suspicious nodes in bilateral cervical VI, left level III, and IV nodes. In view of high serum Tg, [18F]FDG-PET/CT was done, which revealed hypermetabolic cervical, mediastinal, and right hilar nodes. Fine-needle aspiration cytology (FNAC) from the right level VI node showed metastatic PTC. Surgery was deferred, and the patient was kept on follow-up under thyroxine suppression in view of subcentimetric size of cervical nodes. The patient denied radiofrequency ablation and followed up 6-monthly intervals.

**Fig. 1:**
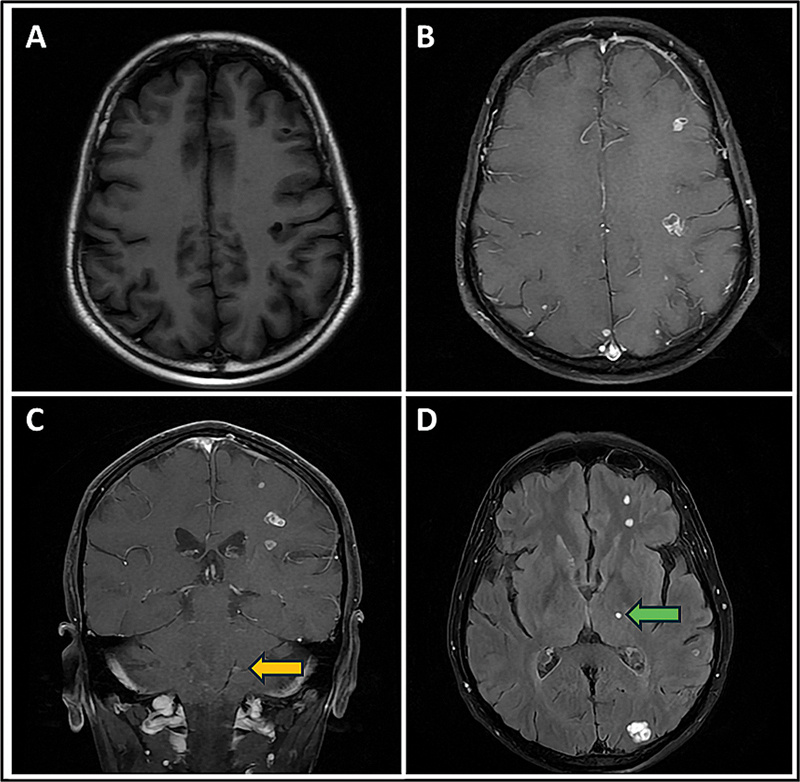
Axial T1-weighted image ( **A** ) showing hypointense lesions in the left cerebral parenchyma. Axial ( **B** ) and coronal ( **C** ) T1-weighted + CE images showing contrast enhancement in the lesions involving the left cerebrum and left cerebellum (yellow arrow). Axial T2-weighted FLAIR + CE ( **D** ) showing enhancing lesions in the left cerebral parenchyma and left thalamus (green arrow). CE, contrast-enhanced; FLAIR, fluid-attenuated inversion recovery.

**Fig. 2:**
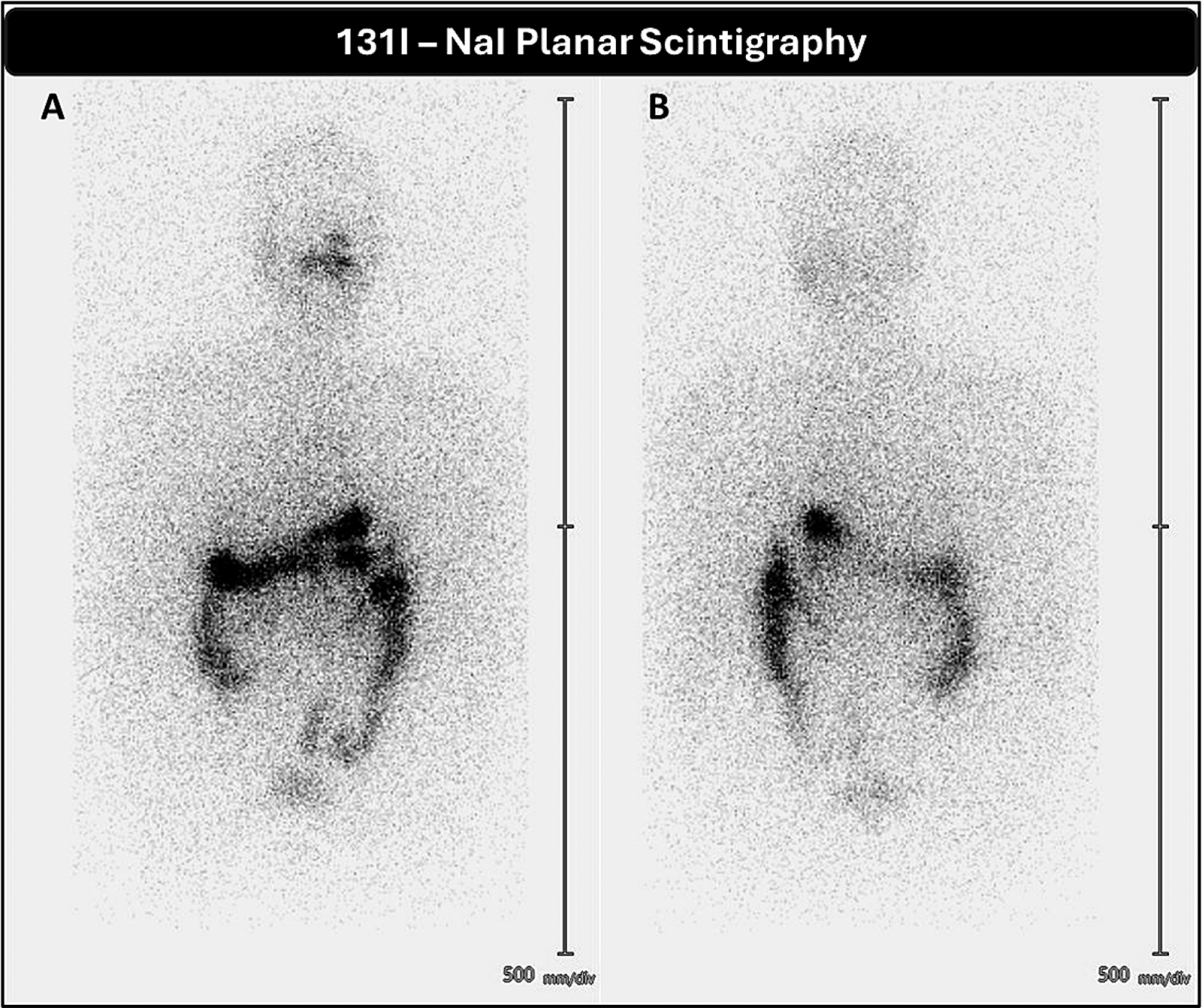
Anterior ( **A** ) and posterior ( **B** ) projections of [ ^131^ I]-NaI planar scintigraphy demonstrating no abnormal radioiodine concentration in the whole-body survey except for physiological uptake in nasopharyngeal mucosa and gut.

**Fig. 3:**
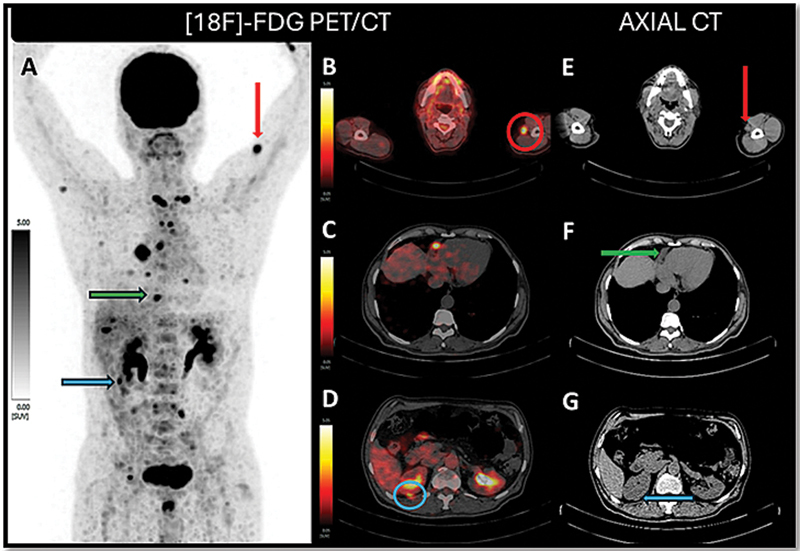
MIP of [ ^18^ F]FDG-PET/CT ( **A** ) showing extensive metastatic disease involving cervical and mediastinal nodes, skeleton, liver, subcutis of left arm (red arrow), pericardium (green arrow), and kidneys (blue arrow). The second column showing axial fused [18F]FDG-PET/CT images showing FDG-avid subcutaneous nodule in the left arm (red circle) ( **B** ), pericardial deposit ( **C** ), and right kidney cortical deposit (blue circle) ( **D** ). The third column shows representative axial CT sections showing a subcutaneous nodule of the left arm (red arrow) ( **E** ), pericardial deposit (green arrow) ( **F** ), and right kidney cortical deposit (blue arrow) ( **G** ). CT, computed tomography; FDG, fluorodeoxyglucose; MIP, maximum intensity projection; PET, positron emission tomography.

After 2 years of follow-up, he showed a mild increase in size of cervical nodes and serum Tg of 316 ng/mL. He was clinically suspected to have developed brain metastasis. Contrast-enhanced magnetic resonance imaging (MRI) revealed multiple (at least 25) solid, variably sized, enhancing metastatic lesions involving the bilateral cerebral hemispheres, cerebellum, and left thalamus ( Fig. 1 ). The patient received 10 fractions of whole-brain radiotherapy (WBRT) cumulating to a dose of 30 Gy. He was further evaluated for further RAI therapy and showed elevated serum Tg of > 350 ng/mL and negative iodine scintigraphy. However, he received a trial of empirical RAI therapy (7.1 GBq). Posttherapy and follow-up RAI planar scintigraphy showed no abnormal iodine concentration ( Fig. 2 ) with elevated serum Tg level of > 300 ng/mL. [18F]FDG-PET/CT done for restaging revealed extensive metastatic disease ( Figs. 3 4 5 ) involving cervical and mediastinal lymph nodes (maximum standardized uptake value [SUV _max_ ]: 10.5), axial skeleton (SUV _max_ : 4.37), liver (SUV _max_ : 7.1), bilateral kidneys (SUV _max_ : 5.9), pericardium (SUV _max_ : 6.84), right triceps brachii muscle (SUV _max_ : 3.04), and left arm subcutaneous nodule (SUV _max_ : 8.75). FNAC of left arm subcutaneous nodule was positive for malignancy consistent with metastatic papillary carcinoma of thyroid. Next-generation sequencing was uninterpretable due to suboptimal library quality.

**Fig. 4:**
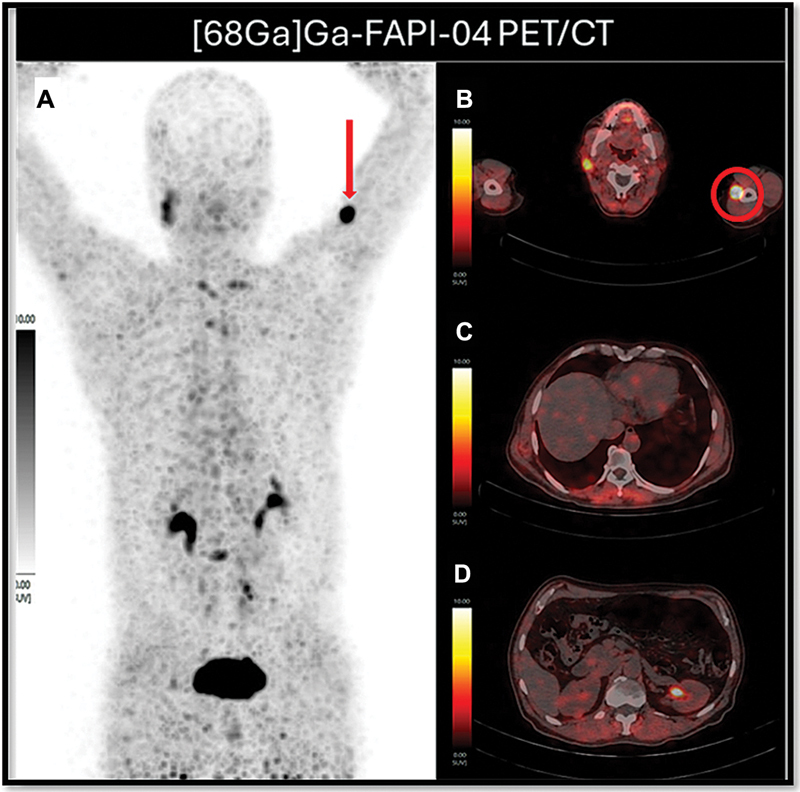
MIP of [68Ga]-FAPI-04 PET/CT ( **A** ) showing intense FAP expression in the subcutaneous nodule of the left arm (red arrow) and nil to low FAP expression at the remaining metastatic sites. Right-sided column shows axial fused [68Ga]-FAPI-04 PET/CT images with intensely FAP expressing subcutaneous nodule (red circle) ( **B** ), non-FAP expressing pericardial deposit ( **C** ), and non-FAP expressing right kidney cortical deposit ( **D** ). CT, computed tomography; FAP, fibroblast activation protein; FAPI, fibroblast activation protein inhibitor; MIP, maximum intensity projection; PET, positron emission tomography.

**Fig. 5:**
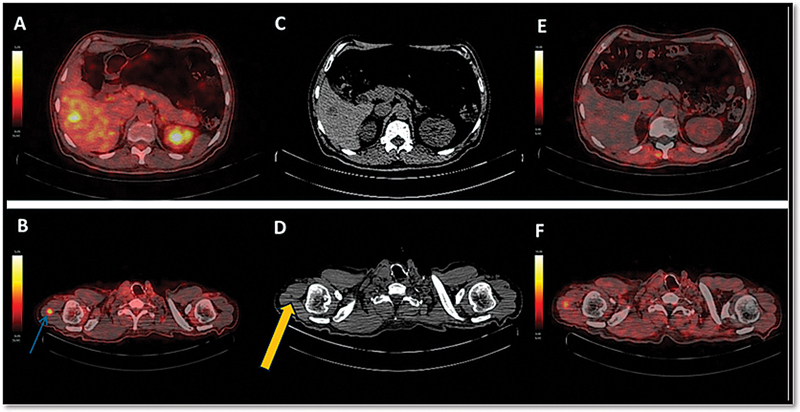
The first column showing axial fused [ ^18^ F]FDG-PET/CT images showing FDG concentrating liver metastasis in segment VI of the liver ( **A** ) and metastasis in the right triceps muscle (blue arrow) ( **B** ). The second column showing representative axial CT sections showing a hypodense lesion in segment VI of the liver ( **C** ) and hypodense lesion in the right triceps muscle (yellow arrow) ( **D** ). The third column showing axial fused [68Ga]Ga-FAPI-04 PET/CT demonstrating no FAP expression in the segment VI liver lesion ( **E** ) and low FAP expression (SUV _max_ : 4.3) in the right triceps muscle metastasis ( **F** ). CT, computed tomography; FAP, fibroblast activation protein; FAPI, fibroblast activation protein inhibitor; FDG, fluorodeoxyglucose; PET, positron emission tomography; SUV _max_ , maximum standardized uptake value.

The patient underwent [68Ga]Ga-FAPI-04 PET/CT ( Figs. 3 4 5 ). The FAPI-04 molecule was procured from MedChemExpress, and [68Ga]Ga-FAPI-04 was prepared in an in-house automated module to evaluate for fibroblast activation protein (FAP) expression and therapeutic feasibility which revealed nil to very low-grade FAP expression at the metastatic sites except for few cervical and mediastinal nodes (SUV _max_ : 8.2), left arm subcutaneous nodule (SUV _max_ : 26.7), and L3 vertebral lytic lesion (SUV _max_ : 10.4). The comparative SUV _max_ values on [18F]FDG-PET/CT and [68Ga]Ga-FAPI-04 PET/CT are depicted in Table 1 . The patient was interpreted as not a candidate for targeted radionuclide therapy. He was referred to medical oncology for oral tyrosine kinase inhibitors (TKIs) and started on lenvatinib 24 mg OD. He is currently faring well with no adverse effects.

**Table 1:** Comparison of SUV _max_ values on FDG-PET and FAPI PET across sites in the studied patient

Lesion site	FDG SUV _max_	FAPI SUV _max_	Interpretation
Cervical and mediastinal lymph nodes	10.5	8.2	Both avid, FDG > FAPI
Axial skeleton (L3 vertebral lesion)	4.37	10.4	FAPI > FDG—desmoplastic lesion
Liver (segment VI)	7.1	Nil	FDG positive, FAPI negative
Bilateral kidneys	5.9	Nil	FDG positive, FAPI negative
Subcutaneous metastasis	8.75	26.7	FAPI > FDG—FAP-dominant lesion, fibroblast-rich stroma
Pericardium	6.84	Nil	FDG positive, FAPI negative
Right triceps muscle	3.04	4.3	Mild uptake on both, FAPI slightly > FDG
Average SUV _max_ (across lesions)	6.92	8.77	Predominantly FDG-avid disease overall with focal FAPI-dominant lesions

Abbreviations: FAP, fibroblast activation protein; FAPI, fibroblast activation protein inhibitor; FDG, fluorodeoxyglucose; PET, positron emission tomography; SUV _max_ , maximum standardized uptake value.

## Discussion

TENIS is not an uncommon entity with a reported incidence of 2 to 27% in the literature. 1 2 Outcome is poor in these patients, with 10-year survival rates usually less than 10% and a mean life expectancy of 3 to 5 years. 7 Distant metastatic disease is an important predictor of survival in thyroid cancer and is associated with unfavorable outcomes. 2 3 Common site of distant metastasis from thyroid cancer includes the lungs and skeleton, which tend to be RAIR. 3 8 9 TENIS is highly prevalent in PTC, and genomic predictors for developing TENIS include BRAFV600E and TERT promoter mutations. 8 10

Distant metastasis excluding lung and skeletal metastasis and multiple organ involvement is rare in thyroid carcinoma and the presence of widespread distant metastases indicates a poor clinical outcome. 4 Brain metastasis is seen in around 5% of thyroid cancer patients and has a significant impact on overall survival rates, in addition to multiorgan involvement and male gender. 9 Early detection and aggressive treatment of brain metastasis is recommended, and therapeutic options are surgery followed by WBRT or stereotactic radiosurgery (SRS), followed by WBRT for patients who are newly diagnosed and have stable systemic disease, while for patients with more than three metastatic lesions in the brain, SRS or WBRT is advisable, as is the case in our patient. 9 11

Incidence of cardiac metastasis ranges between 2.3 and 18.3% across different primary malignancies, with only a handful reported from thyroid cancer. 12 13 14 15 Common presentation includes shortness of breath, hypotension, tachycardia, and features of tamponade in pericardial involvement; arrhythmias, AV blocks, conduction disturbances, heart failure, and myocardial infarction in myocardial involvement. Prognosis is usually dismal, and the therapeutic strategy is based on the surgical feasibility and the primary malignancy. 16

Renal metastasis from thyroid cancer is rare and clinical prevalence was estimated to be between 0.47 and 5.3%, usually in cases of follicular thyroid carcinoma with extensive multiple sites of metastasis. Minor venous plexuses and lymphatic collaterals are thought to be a plausible cause for renal metastasis from the thyroid. 17 18 19 Cutaneous metastasis from thyroid carcinoma is rare and usually occurs in the setting of disseminated neoplastic disease, with papillary carcinoma being the most common type and scalp being the most common site for metastasis. 20 21 Skeletal muscle metastasis from thyroid carcinoma is extremely rare, with a little above 50 cases reported in the literature and is associated with unfavorable survival outcomes. The most common site of metastasis was the gluteus muscle, and the majority were caused by PTC. 22 23

TENIS puts treating physicians in a quandary whether to consider empirical RAI therapy or otherwise. National Comprehensive Cancer Network and ATA conditionally recommend empirical RAI therapy in patients with negative iodine scans with rapidly rising Tg levels, previous response to RAI, and in tumors not amenable to local therapy. 3 24 Although this demonstrates a fall in Tg in more than half of the patients, no significant benefit has been noted in overall survival. 3 In the case of our patient, there was neither biochemical nor structural response to empirical RAI therapy.

[18F]FDG-PET/CT plays a crucial role in TENIS for prognostication, risk stratification, and treatment guidance. 1 It is currently conditionally recommended by the ATA in cases of DTC (aggressive variants) with elevated serum Tg and/or negative iodine imaging. Meta-analysis of 17 studies revealed a pooled sensitivity and specificity of 86 and 84%, respectively. 3 [18F]-FDG-PET/CT-derived parameters, mainly uptake values, have prognostic significance in DTC patients. 25 For brain metastasis, [18F]-FDG-PET/CT has the inherent disadvantage of high physiological uptake in the brain, and MRI remains the preferred modality.

FAPI-PET/CT, a novel imaging modality, has shown promising results, making it a valid alternative to [18F]-FDG PET/CT for thyroid cancer. This is particularly true for aggressive variants and RAIR cases, where existing treatment options are limited. 5 6 26 27 Contrary to these results, our case showed no significant FAPI expression at the metastatic sites except for a few named ones as mentioned earlier (subcutaneous and skeletal metastasis), rendering unsuitable for targeted radionuclide therapy. Interestingly, it was noted that subcutaneous metastasis demonstrated intense FAP expression (SUV _max_ : 26.7) as compared with FDG uptake (SUV _max_ : 8.75). A case of cutaneous metastasis from ovarian cancer has been reported with intense FAP expression. 28 Further larger cohort studies are recommended to comment on the performance and uptake patterns of FAPI in both cutaneous primaries and metastasis.

Oral TKIs remain the approved treatment modalities in progressive and extensive TENIS/RAIR-DTC patients, and lenvatinib remains the initial choice due to its multikinase inhibitor action resulting in better response rate and progression-free survival. 29 30 Our case shows heterogeneity within patients between metabolic activity (FDG) and stromal FAP expression (FAPI). The discordance suggests that FDG-avid disease does not always come with a cancer-associated fibroblast–rich or desmoplastic microenvironment. As a result, not all FDG-positive lesions will be appropriate targets for FAP-directed radionuclide therapy. Practically, this means we should perform lesion-level FAPI imaging and, when possible, correlate it with biopsy, immunohistochemistry, and dosimetry when considering FAPI-based treatments. Heterogeneous FAP expression may reduce whole-body therapeutic effectiveness and support a more tailored or hybrid approach. This could involve local therapy or systemic TKI for FAPI-negative disease and radioligand therapy for truly FAPI-positive deposits.

## Conclusion

In this report, we presented an extremely rare case of TENIS with extensive multiple UDM, and comparative evaluation of [18F]-FDG and [68Ga]Ga-FAPI-04 PET/CT uptake patterns at metastatic sites, which showed superior performance of [18F]-FDG in this case. Of note, intense FAP expression has been noted in the metastatic subcutaneous deposit as compared with FDG uptake, and this heterogeneity could be an area of research to define uptake patterns of FAPI in cutaneous and subcutaneous malignancies. Thus, [68Ga]Ga-FAPI-04 PET/CT may be useful in selected cases and metastatic sites.
